# Osteocyte ferroptosis induced by ATF3/TFR1 contributes to cortical bone loss during ageing

**DOI:** 10.1111/cpr.13657

**Published:** 2024-05-19

**Authors:** Ying Yin, Guang‐Jin Chen, Chen Yang, Jia‐Jia Wang, Jin‐Feng Peng, Xiao‐Fei Huang, Qing‐Ming Tang, Li‐Li Chen

**Affiliations:** ^1^ Department of Stomatology, Union Hospital, Tongji Medical College Huazhong University of Science and Technology Wuhan China; ^2^ School of Stomatology, Tongji Medical College Huazhong University of Science and Technology Wuhan China; ^3^ Hubei Province Key Laboratory of Oral and Maxillofacial Development and Regeneration Wuhan China

## Abstract

Cortical bone loss is intricately associated with ageing and coincides with iron accumulation. The precise role of ferroptosis, characterized by iron overload and lipid peroxidation, in senescent osteocytes remains elusive. We found that ferroptosis was a crucial mode of osteocyte death in cortical bone during ageing. Using a single‐cell transcriptome analysis, we identified activating transcription factor 3 (ATF3) as a critical driver of osteocyte ferroptosis. Elevated ATF3 expression in senescent osteocytes promotes iron uptake by upregulating transferrin receptor 1 while simultaneously inhibiting solute carrier family 7‐member 11‐mediated cystine import. This process leads to an iron overload and lipid peroxidation, culminating in ferroptosis. Importantly, ATF3 inhibition in aged mice effectively alleviated ferroptosis in the cortical bone and mitigated cortical bone mass loss. Taken together, our findings establish a pivotal role of ferroptosis in cortical bone loss in older adults, providing promising prevention and treatment strategies for osteoporosis and fractures.

## Graphical abstract

Our research demonstrated ferroptosis was the crucial mode of osteocyte death in cortical bone during ageing, in which ATF plays a key role. ATF3 in senescent osteocytes promotes iron uptake via up‐regulating transferrin receptor 1 (TFR1) while simultaneously inhibiting solute carrier family 7a member 11‐mediated cystine import. This process leads to iron overload, lipid peroxidation and culminates in ferroptosis, osteocyte death and cortical bone loss.

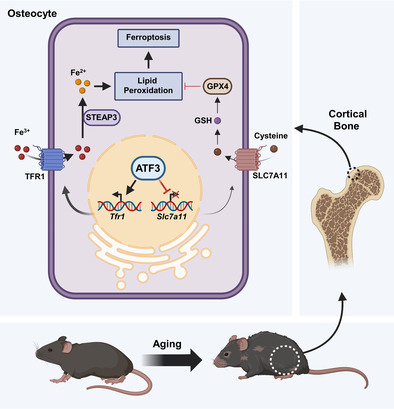

## 1 INTRODUCTION

Bone ageing is a complex process characterized by a progressive decline in bone mass and density, leading to the development of age‐associated osteoporosis. The estimated lifetime risk of osteoporotic fracture is one in three for women and one in five for men aged >50 years.^1^, ^2^, ^3^, ^4^ This fracture risk, especially in older adults who already have poor health, contributes significantly to healthcare costs and poses a substantial public health concern.^5^ Bone ageing manifests in various ways, including osteocyte death, diminished trabecular bone quantity and thickness and cortical bone thinning.^5^, ^6^, ^7^, ^8^, ^9^ Cortical bone thinning is associated with a higher risk of fracture than trabecular bone loss, highlighting the clinical importance of addressing this loss.^1^, ^2^, ^3^, ^4^ Although both cortical and trabecular osteocytes originate from osteoblasts, they exhibit distinct characteristics and developmental pathways.^10^, ^11^, ^12^, ^13^, ^14^ In addition, cortical and trabecular bones differ in their fate during ageing.^15^ However, the mechanisms underlying cortical bone loss during ageing remain unclear.

During age, the loss of cortical bone is mainly due to the increase in many osteoclasts around the cortical bone and the absorption at the endocortical surface and the amount of bone absorption exceeds that of osteogenesis on the surface of the periosteum.^16^ Following osteocyte death, neighbouring osteocytes are stimulated to secrete receptor activator of nuclear factor kappa‐B ligand (RANKL), a mediator that recruits osteoclasts to degrade and absorb bone matrix.^17^, ^18^, ^19^, ^20^ Mice lacking RANKL in osteocytes had fewer pericortical osteoclasts and more bone loss as they aged than control mice.^21^ In addition, osteocytes can secrete osteoprotegerin, a RANKL inhibitor, and macrophage colony‐stimulating factor, a cytokine essential for osteoclast differentiation, to regulate osteoclast differentiation. Homeostasis of osteocytes and osteoclasts is a part of bone remodelling. The slower remodelling rate and prolonged lifespan of cortical osteocytes compared to those of trabecular osteocytes inevitably lead to cellular senescence during ageing.^15^ Senescent cells are susceptible to various modes of cell death, including apoptosis, necroptosis, ferroptosis, autophagy, pyroptosis and others.^22^, ^23^, ^24^, ^25^, ^26^, ^27^ Sub‐lethal mitochondrial apoptotic stress is a major driver of senescence‐associated secretory phenotype.^28^ Senescent cells exhibit significantly higher iron ion levels than non‐senescent cells, potentially triggering lipid redox imbalance and ferroptosis.^29^, ^30^ Necroptosis is implicated in age‐dependent brain degeneration and disrupts hippocampal neuronal connectivity and cognitive function.^27^ Ferroptosis, a non‐apoptotic cell death mechanism triggered by iron dyshomeostasis, and its molecular mechanisms include transferrin receptor 1 (TFR1), solute carrier family 7a member 11 (SLC7A11) and various transcription factors such as activating transcription factor 3 (ATF3). Increasing evidence suggests that ferroptosis plays an important role in the ageing process of the body.^31^ However, the precise mechanism underlying osteocyte death during ageing remains unclear.

In this study, we revealed that a significant surge in osteocyte ferroptosis is a key cause of age‐related cortical bone mass loss. With ageing, the excess accumulation of Fe ions within osteocytes induces lipid peroxidation, culminating in cell death. In addition, we used small molecule compounds to inhibit ferroptosis and evaluated their efficacy in mitigating cortical bone loss and restoring bone health. Overall, this study has immense potential for improving bone health, combating age‐related bone loss, preventing fractures and ultimately enhancing the quality of life of old people.

## 2 RESULTS

### 2.1 Age‐related increases in cortical osteocyte death occur concurrently with a significant rise in ferroptosis

**FIGURE 1:**
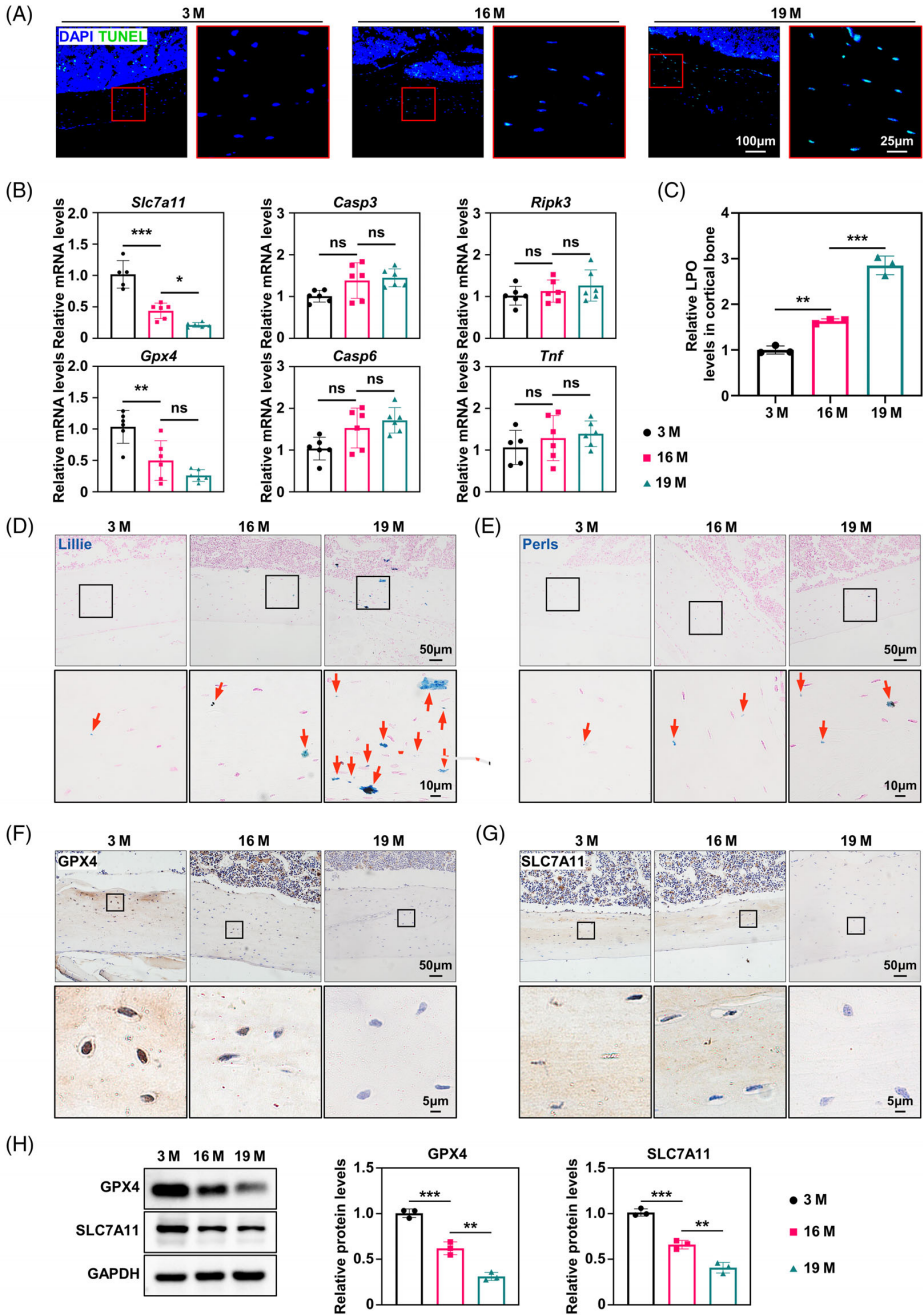
Age‐related increases in cortical osteocyte death occur concurrently with a significant rise in ferroptosis. (A) Representative terminal deoxynucleotidyl transferase dUTP nick end labelling (TUNEL) staining of femur cortical bone from 3‐, 16‐ and 19‐month‐old mice (*n* = 3). (B) Quantitative reverse transcriptase PCR (qRT‐PCR) analysis of the levels of solute carrier family 7a member 11 (*Slc7a11*), glutathione peroxidase 4 (*Gpx4*), *Casp3*, *Casp6*, *Ripk3* and *Tnf* in the femur cortical bone (*n* = 3). (C) Lipid peroxide colorimetric assay of femur cortical bone (*n* = 3). (D) Representative Lillie staining (blue) of the femur cortical bone, showing ferrous iron content in the cortical bone (*n* = 3). (E) Representative Perl's iron staining (blue) of the femur cortical bone, showing the ferric iron content in the cortical bone (*n* = 3). (F) Immunohistochemical staining for GPX4 in the femur cortical bone (*n* = 3). (G) Immunohistochemical staining for SLC7A11 in the femur cortical bone (*n* = 3). (H) Western blot analysis of the levels of GPX4 and SLC7A11 in the femur cortical bone, with quantitative data on the right (*n* = 3). M, months. *p < 0.05, **p < 0.01, ***p < 0.001.

A series of assays were performed on cortical bone tissues to elucidate the mode of osteocyte death during cortical bone ageing. However, terminal deoxynucleotidyl transferase dUTP nick end labelling (TUNEL) staining unequivocally demonstrated an age‐dependent increase in osteocyte death within the cortical bone (Figure 1A). In addition, expression of ferroptosis‐associated markers, *Slc7a11* and glutathione peroxidase 4 (*Gpx4*), showed a significant upregulation with advancing age, whereas apoptosis (caspase 3 [*Casp3*] and caspase 6 [*Casp6*]) and necroptosis (receptor‐interacting serine–threonine kinase 3 [*Ripk3*] and tumour necrosis factor [*Tnf*]) genes remained unaltered in the cortical bone during ageing (Figure 1B), suggesting that ferroptosis might be the predominant mechanism underlying osteocyte death during cortical bone ageing. Our investigation of lipid peroxides, the most critical indicator of ferroptosis, within the femoral cortical bone of mice aged 3, 16 and 19 months revealed a significant age‐related increase in ferroptosis activity (Figure 1C). Lillie and Prussian blue staining revealed a marked elevation in iron content, encompassing both ferrous (Fe^2+^) and ferric (Fe^3+^) forms, within the cortical osteocytes of aged mice (Figure 1D,E). Interestingly, no iron staining was observed in the trabecular bone (Figure S1A,B), implying reduced susceptibility to ferroptosis in the trabecular bone compared with that in the cortical bone. We also examined the expression patterns of GPX4 and SLC7A11, which are critical regulatory molecules in ferroptosis‐associated antioxidant systems. Immunohistochemical staining and western blotting demonstrated substantial downregulation of both GPX4 and SLC7A11 in femur osteocytes of 16‐ and 19‐month‐old mice (Figure 1F–H). Collectively, these data provide compelling evidence for the activation of ferroptosis in the cortical osteocytes of aged mice, strongly suggesting that ferroptosis may be the primary mode of osteocyte death during age‐related cortical bone loss.

### 2.2 ATF3 is essential for ferroptosis of cortical osteocytes in aged mice

**FIGURE 2:**
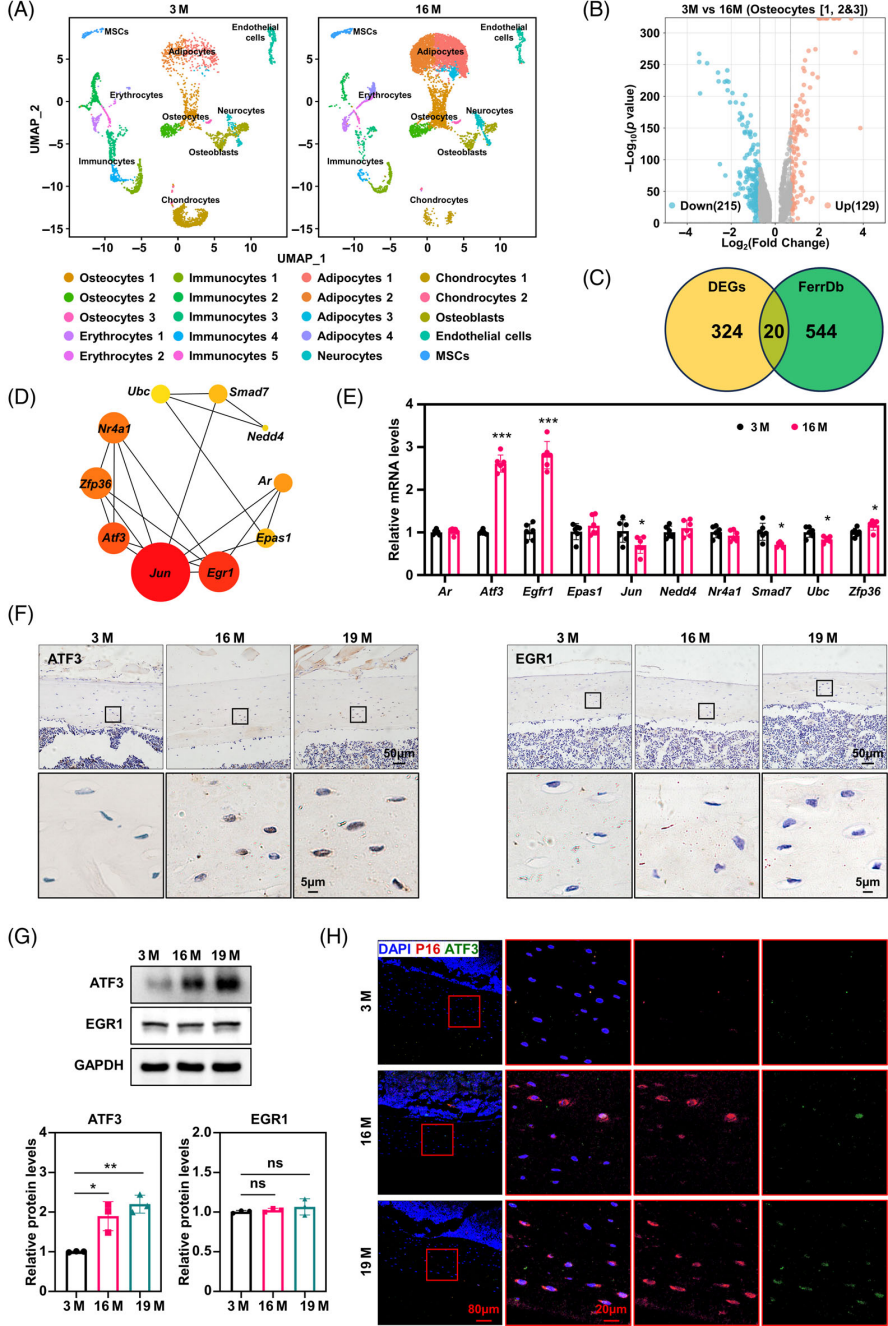
Activating transcription factor 3 (ATF3) is essential for ferroptosis of cortical osteocytes in aged mice (A). The UMAP (uniform manifold approximation and projection) plot of long bone cells from 3‐ and 16‐month‐old mice. The original data comes from Gene Expression Omnibus (GSE145477). (B) Volcano plot showing the differentially expressed genes (DEGs) of osteocyte clusters in (A) from 3‐ and 16‐ month‐old mice. Genes were listed in Table S2. (C) Venn diagram showing DEGs in a public dataset (FerrDb) about ferroptosis‐related genes. Genes were listed in Table S3. (D) Gene network revealing top 10 DEGs scored by Cytoscape software using cytoHubba plugin. The scores of the 10 DEGs are listed in Table S4. (E) Confirmation of the top 10 DEGs using qRT‐PCR (*n* = 3). */**/***Compared with 3 months. (F) Immunohistochemical staining for ATF3 and EGR1 in femur cortical bone of 3‐, 16‐ and 19‐month‐old mice (*n* = 3). (G) Western blot analysis of the levels of ATF3 and EGR1 in the femur cortical bone, with quantitative data below (*n* = 3). (H) Immunofluorescent co‐localization staining for P16 and ATF3 in the femur cortical bone (*n* = 3). M, months. *p < 0.05, **p < 0.01, ***p < 0.001.

To identify the key ferroptosis‐associated genes involved in bone senescence, we conducted a bioinformatics analysis of the transcriptomes of 13,629 individual cells using single‐cell sequencing data from the Gene Expression Omnibus database (GSE145477). Cells from the long bones of 3‐ and 16‐month‐old mice were clustered together based on their transcriptional profiles using unsupervised principal component analysis and visualized using a nonlinear dimensionality‐reduction technique, uniform manifold approximation and projection (Figure 2A). Osteocytes formed different sub‐clusters, characterized by differential expression of osteogenic differentiation genes such as *Runx2* (runt‐related transcription factor 2), *Alpl* (alkaline phosphatase), *Col1a1* (collagen alpha‐1[I] chain), *Sp7* (transcription factor Sp7), *Bglap* (osteocalcin), *Ibsp* (integrin‐binding sialoprotein) and *Dmp1* (dentin matrix acidic phosphoprotein 1), indicating that different subgroups are at different stages of osteogenic differentiation (Figure S2A). Here, we extracted differentially expressed genes (DEGs) in all osteocyte clusters (1989 individual cells), and a volcano plot showed that there are 129 upregulated genes and 215 down‐regulated genes in osteocytes between 3‐ and 16‐month‐old mice (Figure 2B). Notably, 20 genes overlapped between DEGs and ferroptosis‐related genes (FerrDb database) (Figure 2C). We constructed a protein–protein interaction network of hub ferroptosis‐related DEGs, including androgen receptor, *Atf3* (activating transcription factor 3), *Egr1* (early growth response 1), *Epas1* (endothelial PAS domain protein 1), *Jun* (Jun proto‐oncogene), *Nedd4* (neural precursor cell expressed, developmentally down‐regulated 4), *Nr4a1* (nuclear receptor subfamily 4 group A member 1), *Smad7* (SMAD family member 7), *Ubc* (ubiquitin C) and *Zfp36* (Zinc finger protein 36) (Figure 2D). To validate our bioinformatics results, we evaluated the expression levels of these hub ferroptosis‐related DEGs. The results showed that *Atf3* and *Egfr1* were significantly upregulated by twofold to threefold in the osteocytes of 16‐month mice femurs (Figure 2E). However, only the protein levels of ATF3 increased in the femurs of 19‐month mice (Figure 2F,G). By the way, we did not see age‐specific differences in ATF3 expression in trabecular osteocytes (Figure S2B). Subsequent cortical bone immunofluorescence co‐localization staining showed that ATF3 was mostly expressed in osteocytes with high levels of the senescence markers, P16 (Figure 2H). In addition, single‐cell data analysis showed that ATF3 expression showed an age‐related increase in different clusters, and the cluster (osteocytes 3) with higher ATF3 expression also had higher expression of age‐related genes, such as *Cdkn1a* (cyclin‐dependent kinase inhibitor 1), *Tgfb1* (transforming growth factor beta‐1 proprotein), *Serpine1* (plasminogen activator inhibitor 1) and *Cdkn2a* (cyclin‐dependent kinase inhibitor 2A) (Figure S2C). These results suggest that ATF3 may be a key ferroptosis‐driven gene that regulates osteocyte ferroptosis in aged cortical bone tissues.

### 2.3 ATF3‐induced ferroptosis in senescent osteocytes is not primarily mediated by SLC7A11

**FIGURE 3:**
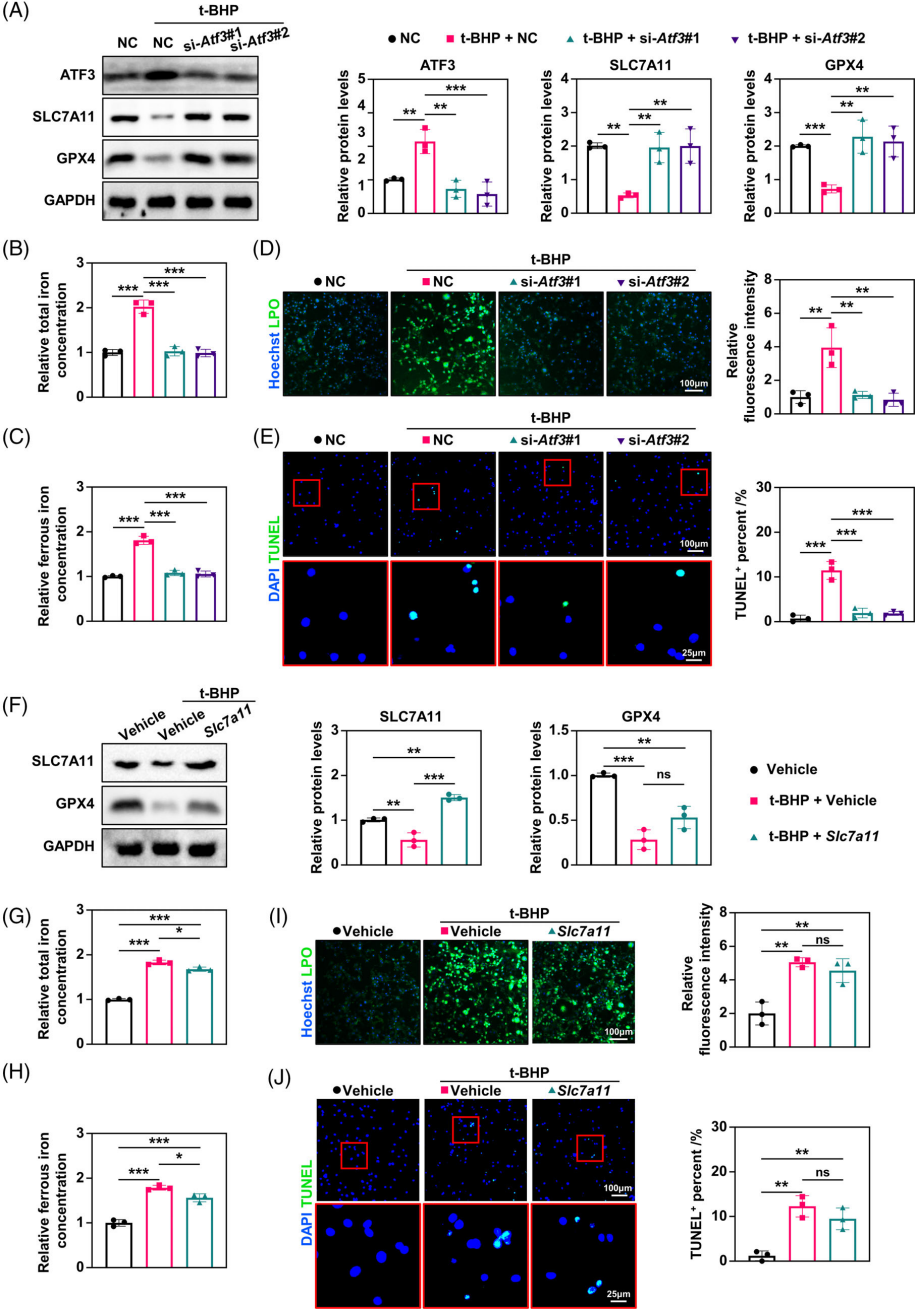
Activating transcription factor 3 (ATF3)‐induced ferroptosis in senescent osteocytes is not primarily mediated by solute carrier family 7a member 11 (SLC7A11). (A) Western blot analysis of the levels of ATF3, SLC7A11 and glutathione peroxidase 4 (GPX4) in MLOY4 cells at indicated treatment, with quantitative data at right (*n* = 3). (B and C) Total iron and ferrous iron levels in MLOY4 cells corresponding to the samples in A were quantitatively determined using iron assay kits (*n* = 3). (D) Representative immunofluorescence of lipid peroxidation (LPO) levels in MLOY4 cells at indicated treatment by using LPO Fluorometric assay kits, with quantitative data on the right (*n* = 3). (E) Representative images of the terminal deoxynucleotidyl transferase dUTP nick end labelling (TUNEL) assay in MLOY4 cells at indicated treatment, with quantitative data on the right (*n* = 3). (F) Western blot analysis of the levels of SLC7A11 and GPX4 in MLOY4 cells at indicated treatment, with quantitative data on the right (*n* = 3). (G and H) Total iron and ferrous iron levels in MLOY4 cells corresponding to the samples in F were quantitatively determined using iron assay kits (*n* = 3). (I) Representative immunofluorescence of LPO levels in MLOY4 cells at indicated treatment by using LPO Fluorometric assay kits, with quantitative data on the right (*n* = 3). (J) Representative images of the TUNEL assay in MLOY4 cells at indicated treatment, with quantitative data on the right (*n* = 3). *p < 0.05, **p < 0.01, ***p < 0.001.

To further explore the role of ATF3 in ferroptosis in senescent osteocytes, we down‐regulated the expression of *Atf3* by siRNA transfection in MLOY4 osteocytes and analysed iron metabolism and lipid peroxidation. Literature suggests that ATF3 induces ferroptosis by inhibiting SLC7A11.^32^ We found that *Atf3* downregulation increased the expression of SLC7A11 and GPX4 in MLOY4 cells treated with tert‐Butyl hydroperoxide (t‐BHP), a classic inducer of cell senescence (Figure 3A). In addition, *Atf3* downregulation effectively reduced the total cellular iron levels, lipid hydroperoxide and cell death in MLOY4 cells treated with t‐BHP (Figure 3B–E). However, SLC7A11 overexpression partially increased the expression of GPX4 and decreased the levels of cellular iron, lipid hydroperoxide and cell death in MLOY4 cells treated with t‐BHP (Figure 3F–J). However, SLC7A11 overexpression resulted in only a small number of saves. These data demonstrate that ATF3 is a key molecule in regulating ferroptosis in osteocytes, whereas SLC7A11, a downstream target of ATF3, appears to play a less prominent role.

### 2.4 ATF3/TFR1 axis is a primary mediator of iron accumulation and ferroptosis in senescent osteocytes

**FIGURE 4:**
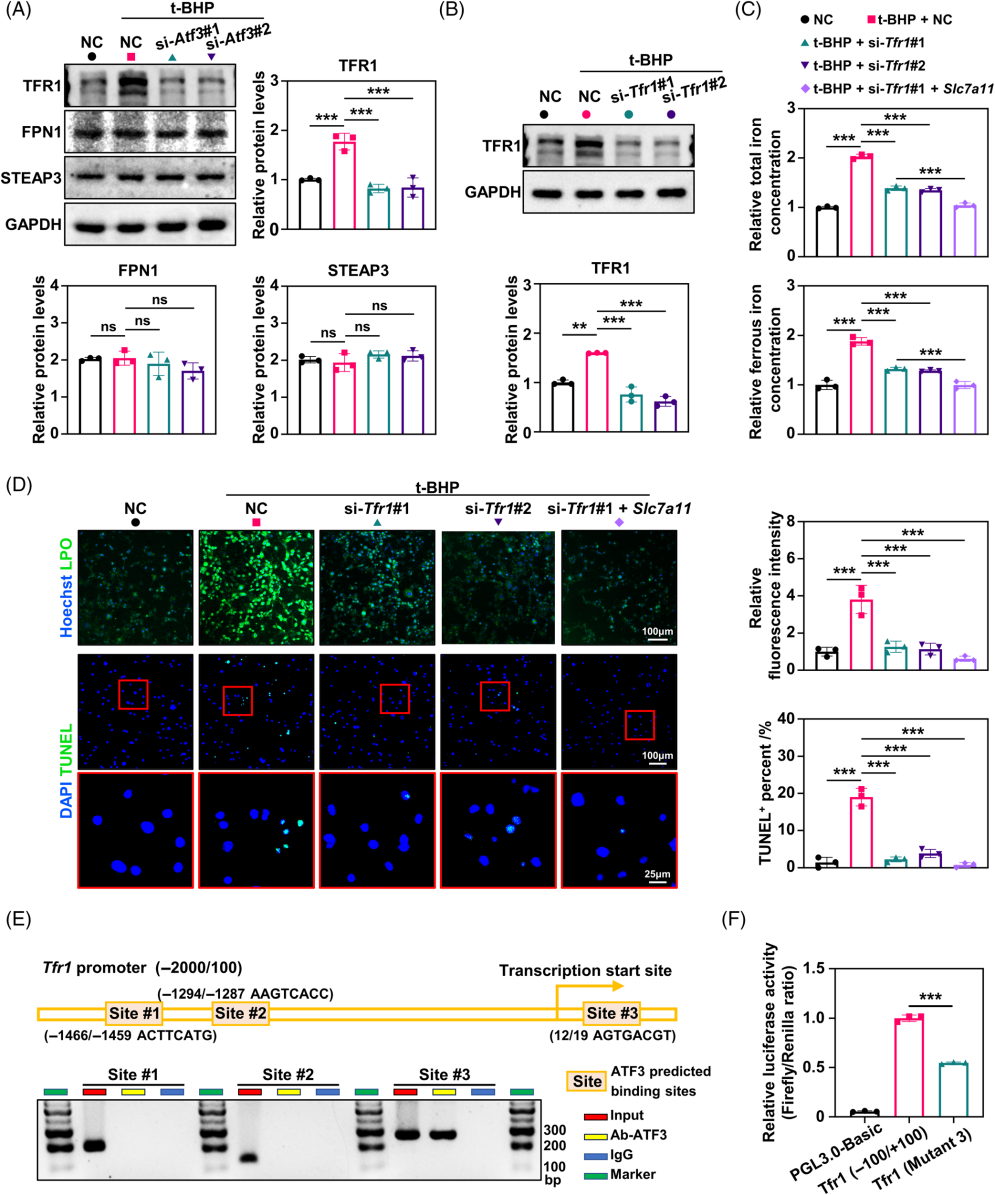
The activating transcription factor 3 (ATF3)‐transferrin receptor 1 (TFR1) axis is a primary mediator of iron accumulation and ferroptosis in senescent osteocytes. (A) Western blot analysis of the levels of TFR1, ferroportin‐1 (FPN1) and six‐transmembrane epithelial antigen of prostate 3 (STEAP3) in MLOY4 cells at indicated treatment, with quantitative data (*n* = 3). Only the top bands of TFR1 protein were evaluated. The lower bands were soluble forms of the TFR1 protein. (B) Western blot analysis of the levels of TFR1 in MLOY4 cells at indicated treatment, with quantitative data below (*n* = 3). (C) Total iron and ferrous iron levels in MLOY4 cells at indicated treatment were quantitatively determined using iron assay kits (*n* = 3). (D) Representative immunofluorescence of lipid peroxidation (LPO) levels in MLOY4 cells at indicated treatment using LPO Fluorometric assay kit, with quantitative data on the right (*n* = 3). Representative images of the terminal deoxynucleotidyl transferase dUTP nick end labelling (TUNEL) assay in MLOY4 at indicated treatment, with quantitative data on the right (*n* = 3). (E) The transcription factor ATF3 was bound to the *Tfr1* promoter in MLOY4 cells. Chromatin immunoprecipitation assays were performed using anti‐IgG as a negative control (*n* = 3). (F) Luciferase reporter assays measuring the activities of the wild‐type or mutated ATF3‐binding site at the *Tfr1* promoter in MLOY4 cells (*n* = 3). *p < 0.05, **p < 0.01, ***p < 0.001.

Given the lack of prior studies on SLC7A11's role in intracellular iron accumulation, we shifted our focus to investigate the potential involvement of ATF3 in iron transport. To elucidate the downstream mechanism of ATF3 in iron accumulation in senescent osteocytes, we detected the expression levels of iron transport‐related proteins, including TFR1, ferroportin‐1 (FPN1) and six‐transmembrane epithelial antigen of prostate 3 (STEAP3). We found that TFR1 was significantly increased in senescent osteocytes and that ATF3 downregulation reduced TFR1 but did not significantly alter FPN1 and STEAP3 (Figure 4A). Subsequently, we performed *Tfr1* knockdown and observed a decrease in total and ferrous iron levels in senescent MLOY4 cells (Figure 4B–D). However, TFR1 knockdown mitigated lipid peroxidation and reduced the number of TUNEL‐positive cells (Figure 4E). In addition, TFR1 knockdown and simultaneous overexpression of SLC7A11 improved ferroptosis in senescent osteocytes (Figure 4C,D).

In addition, ATF3 serves as a transcriptional factor that binds to DNA and contributes to transcriptional regulation.^33^ We found that ATF3 binds to the promoter of *Tfr1* (Figure 4E) and activates its transcription (Figure 4F). Here, we demonstrated that ATF3 primarily increased the accumulation of cellular iron and lipid peroxidation, contributing to ferroptosis by directly promoting *Tfr1* expression.

### 2.5 Preventing ATF3 upregulation in aged mice alleviated ferroptosis‐induced cortical bone loss

**FIGURE 5:**
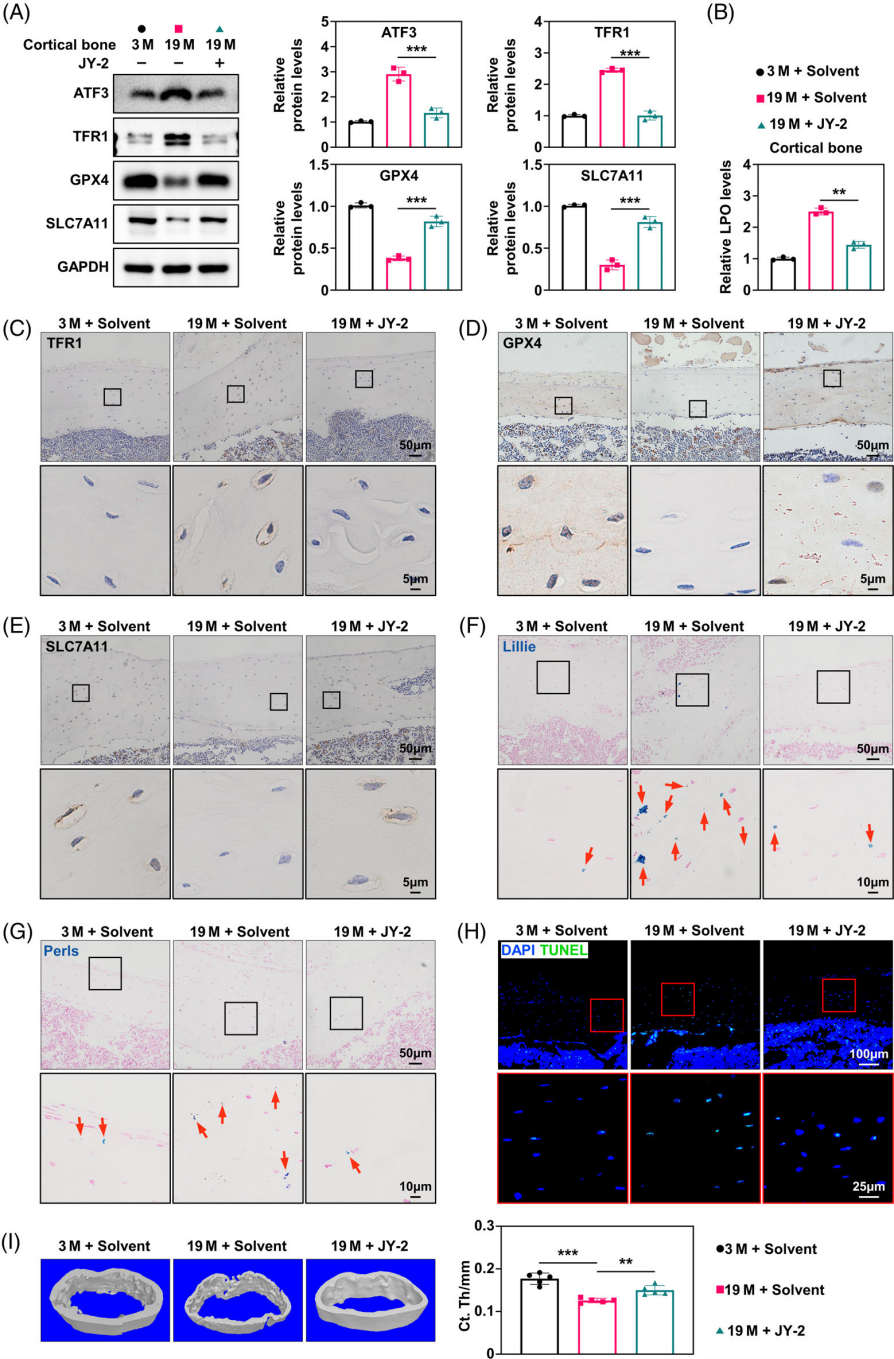
Preventing activating transcription factor 3 (ATF3) upregulation in aged mice alleviated ferroptosis‐induced cortical bone loss. (A) Western blot analysis of the levels of ATF3, transferrin receptor 1 (TFR1), glutathione peroxidase 4 (GPX4) and solute carrier family 7a member 11 (SLC7A11) at indicated time in the femur cortical bone of 3‐ and 19‐month‐old mice treated with solvent or JY‐2 (50 mg/kg/d, p.o.) for four weeks, with quantitative data on the right (*n* = 3). (B). Lipid peroxide colorimetric assay of femur cortical bone (*n* = 3). (C–E) Immunohistochemical staining for TFR1, SLC7A11 and GPX4 of the femur cortical bone (*n* = 3). (F) Representative Lillie staining (blue) of femur cortical bone *n* = 3. (G). Representative Perl's iron staining (blue) of femur cortical bone (*n* = 3). (H) Representative TUNEL staining of femur cortical bone (*n* = 3). (I) Representative images of micro‐CT reconstruction of femoral cortical bone, with quantitative data below (*n* = 5). M, months. *p < 0.05, **p < 0.01, ***p < 0.001.

To test the biological role of ATF3 in vivo, we used JY‐2 to suppress ATF3 expression in 19‐month‐old mice for 4 weeks. Quantitative analysis revealed a substantial decline in ATF3 expression in the femoral cortical bone of the 19‐month‐old mice after JY‐2 administration (Figure 5A). With a decrease in ATF3 expression, TFR1 expression in the cortical bone of the mice also decreased, and GPX4 and SLC7A11 expression increased (Figure 5A). We observed a significant decrease in cortical bone lipid peroxides following JY‐2 treatment in aged mice (Figure 5B). Correspondingly, immunohistochemical results showed that after treatment with JY‐2, TFR1 expression was significantly reduced in the cortical osteocytes of old mice (Figure 5C). Moreover, GPX4 and SLC7A11 protein levels were elevated, and cellular iron concentrations were significantly diminished in the cortical osteocytes of old mice treated with JY‐2 (Figure 5D–G). Notably, JY‐2 effectively reduced the number of TUNEL‐positive cells in the cortical bone and alleviated the senescence‐associated secretory phenotype in cortical bone (Figure 5H and Figure S3A). In addition, the administration of JY‐2 significantly reduced the number of osteoclasts in the medial cortical bone and alleviated age‐related loss of cortical bone mass (Figure 5I and Figure S3B). Taken together, ATF3 inhibition in aged mice can mitigate osteocyte ferroptosis and bone loss in the cortical bone.

## 3 DISCUSSION

This study revealed a novel connection between ageing and ferroptosis in cortical bone. During osteocyte senescence in the cortical bone, the ferroptosis driver, ATF3, underwent significant upregulation. ATF3 directly regulates TFR1 transcription, thereby promoting iron accumulation in senescent osteocytes while simultaneously downregulating the cystine export protein SLC7A11. This convergence of events ultimately leads to iron overload and lipid peroxidation in the osteocytes. In vivo experiments have shown that ATF3 inhibition effectively alleviates iron‐induced cell death and delays cortical bone loss. These findings establish ferroptosis as a crucial fate pathway for osteocytes during cortical ageing and highlight the therapeutic potential of ferroptosis inhibition in mitigating age‐related cortical bone mass loss.

Osteocyte death is an integral component that controls the complex balance of bone formation and resorption.^34^ Andreev et al. found that osteoclasts sensed damage‐associated molecular patterns released by necrotic osteocytes through macrophage induced C‐type lectin, triggering their differentiation and bone loss.^35^ In the context of osteocyte death at different sites, osteoclasts exhibit a higher propensity to resorb osteocytes in areas characterized by low bone density.^36^ Therefore, in the process of natural bone ageing, bone resorption and bone remodelling in the cortical region are slow, and there are a large number of long‐lived osteocytes undergoing ageing. In addition, osteocytes have a longer survival time, with an extraordinary life span of 25 years.^37^ Published studies have shown that Fe ions are more likely to accumulate in ageing tissues and cell bodies.^38^ This phenomenon is consistent with the iron accumulation and lipid peroxidation observed in ageing cortical bone. Subsequently, senescent osteocytes undergo ferroptosis, promoting osteoclast formation and resulting in bone resorption exceeding the amount of new bone formation. In addition, we did not observe significant iron accumulation in the trabecular bone, which may be attributed to frequent bone remodelling and the absence of senescent osteocytes. These findings further emphasize the substantial difference in cortical and trabecular bone loss during the ageing process, and targeting cortical bone loss has greater clinical significance for the prevention and treatment of osteoporotic fractures.

The mechanism of ferroptosis is mainly composed of an iron‐dependent lipid peroxidation system and antioxidant molecular system including GPX4 and SLC7A11, and the balance of these two systems influences cell ageing and death.^39^ GPX4 and SLC7A11 are key proteins in the production of glutathione (GSH), whose function is to inhibit the generation of Fe^2+^‐dependent reactive oxygen species (ROS) by converting lipid hydroperoxides into lipid alcohols.^40^, ^41^ Masaldan et al. found that radiation stimulated MEF senescence and increased TFR1 expression, inducing intracellular Fe^2+^ accumulation.^38^ Zheng et al. found that SLC7A11 expression was down‐regulated in old mouse lung fibroblasts, which hindered Cys transport into the cell and restricted GSH synthesis, increasing intracellular ROS.^42^ Our results suggest that increased expression of age‐related ATF3, a member of the ATF/cAMP‐responsive element binding family of transcription factors, was an important ferroptosis driver in cortical osteocytes. Multiple studies have shown that ATF3 directly regulated SLC7A11 transcription by binding to its promoter region, but this cannot explain the phenomenon of intracellular iron accumulation after elevated ATF3 expression.^32^, ^43^, ^44^, ^45^, ^46^ Our study reveals the unique role of ATF3 in ageing osteocytes. In addition to targeting SLC7A11, ATF3 mainly promotes iron transport into cells by upregulating the expression of TFR1 in osteocytes. Interestingly, ATF3 forms homodimers or heterodimers with other transcription factors, resulting in diverse transcriptional outcomes. Further investigation is warranted to elucidate the precise mechanism by which ATF3 binds to the *Tfr1* promoter region and represses its transcription. On the other hand, Zhang et al. reported that ATF3 was involved in chromatin remodelling and closely related to ageing.^33^ Existing research reports indicate that age‐related inflammatory factors, such as interleukin‐1, interleukin‐6, transforming growth factor‐β and interferon‐γ, promoted ATF3 expression.^47^, ^48^ Additionally, Taketani et al. have reported that p53 directly regulated the expression of ATF3.^49^ In our study, t‐BHP, a conventional cell senescence model construction reagent, was used to induce osteocyte senescence. We found that the expression of ATF3 in osteocytes was upregulated, which is consistent with the conclusions of published studies. In summary, our findings, taken together with those of previously published articles, strongly suggest that the inflammatory microenvironment of ageing and senescent osteocytes leads to an increase in ATF3/TFR1 expression, ultimately leading to ferroptosis in cortical osteocytes.

More and more evidence suggests that ferroptosis has a significant impact on bone metabolism disorders. Jiang et al.'s study suggested that regulating the nuclear factor erythroid 2‐related factor 2/DNA methyltransferases 3A/RANKL axis of osteocytes effectively alleviated the development of postmenopausal osteoporosis by inhibiting ferroptosis.^50^ Xu et al. found that inhibiting ferroptosis in osteoblasts by stimulating the nuclear factor erythroid 2‐related factor 2/GPX4 signalling pathway with vitamin D receptor agonists preferentially hindered age‐related bone loss.^51^ Our study found that ATF3 was the key driving factor for ferroptosis in cortical osteocytes, and inhibiting ATF3‐induced iron accumulation in osteocytes through small molecule JY‐2 effectively reduced the death of senescent osteocytes and alleviated bone loss in mouse cortical bone. Moreover, previous studies have shown that the plasma concentration of JY‐2 after oral administration is higher and more sustained than that after intravenous administration, suggesting that JY‐2 has oral bioavailability.^52^ Our preliminary attempt to target ATF3 to delay age‐related cortical bone mass loss requires further pharmacodynamic and pharmacokinetic optimization, verification of clinical application safety and development of refined application strategies. And, it is necessary to conduct drug modification research on JY‐2 bone targeting.

In conclusion, ferroptosis is the predominant mechanism underlying cortical osteocyte death in older individuals. We demonstrated that ATF3 inhibition can effectively alleviate cortical bone loss in an elderly population. This study reveals a novel therapeutic target for the development of effective interventions against age‐related bone loss.

## 4 MATERIALS AND METHODS

Materials and methods are provided in Supplementary Materials.

## AUTHOR CONTRIBUTIONS

Y. Y., G.‐J.C. and Q.‐M.T. designed the experiments, acquired the data, analysed the data and wrote the manuscript; C.Y. contributed to data acquisition and statistical analyses of the results; J.‐J.W. and J.‐F.P. drafted text for methods and results; X.‐F.H. contributed to data acquisition; L.‐L.C. and Q.‐M.T. were involved in the conception and design of the study, oversaw the completion of studies and substantively revised the manuscript. L.‐L.C. and Q.‐M.T. obtained funding for the project.

## FUNDING INFORMATION

This work is funded by the National Natural Science Foundation of China (U23A20443, 82270950, 82030070) and the Basic Research Support Program of Huazhong University of Science and Technology (2023BR032).

## CONFLICT OF INTEREST STATEMENT

The authors declare no conflicts of interest.

## Supporting information


**Data S1.** Supporting Information.

**Table S2.** DEGs of osteocytes in Figure 2B.
**Table S3.** Overlapping analysis of DEGs and FerrDb.
**Table S4.** Top 10 in network ranked by Maximal Clique Centrality method.

## Data Availability

Data supporting the findings of this study are available from the corresponding author upon request.

## References

[1] Curtis EM , van der Velde R , Moon RJ , et al. Epidemiology of fractures in the United Kingdom 1988‐2012: variation with age, sex, geography, ethnicity and socioeconomic status. Bone. 2016;87:19‐26. 26968752 10.1016/j.bone.2016.03.006 PMC4890652

[2] Kanis JA , Johnell O , Oden A , et al. Long‐term risk of osteoporotic fracture in Malmö. Osteoporos Int. 2000;11(8):669‐674. 11095169 10.1007/s001980070064

[3] Melton LJ III , Chrischilles EA , Cooper C , Lane AW , Riggs BL . Perspective how many women have osteoporosis? J Bone Miner Res. 1992;7(9):1005‐1010. 1414493 10.1002/jbmr.5650070902

[4] Melton LJ III , Atkinson EJ , O'Connor MK , O'Fallon WM , Riggs BL . Bone density and fracture risk in men. J Bone Miner Res. 1998;13(12):1915‐1923. 9844110 10.1359/jbmr.1998.13.12.1915

[5] Sfeir JG , Drake MT , Khosla S , Farr JN . Skeletal aging. Mayo Clin Proc. 2022;97(6):1194‐1208. 35662432 10.1016/j.mayocp.2022.03.011 PMC9179169

[6] Gaumet N , Braillon P , Seibel MJ , Pointillart A , Coxam V , Davicco MJ . Influence of aging on cortical and trabecular bone response to estradiol treatment in ovariectomized rats. Gerontology. 1998;44(3):132‐139. 9592683 10.1159/000021996

[7] Pazzaglia UE , Sibilia V , Congiu T , Pagani F , Ravanelli M , Zarattini G . Setup of a bone aging experimental model in the rabbit comparing changes in cortical and trabecular bone: morphological and morphometric study in the femur. J Morphol. 2015;276(7):733‐747. 25703833 10.1002/jmor.20374

[8] Boonen S , Cheng XG , Nijs J , et al. Factors associated with cortical and trabecular bone loss as quantified by peripheral computed tomography (pQCT) at the ultradistal radius in aging women. Calcif Tissue Int. 1997;60(2):164‐170. 9056165 10.1007/s002239900208

[9] Pathak JL , Bravenboer N , Klein‐Nulend J . The osteocyte as the new discovery of therapeutic options in rare bone diseases. Front Endocrinol (Lausanne). 2020;11:405. 32733380 10.3389/fendo.2020.00405 PMC7360678

[10] Kronenberg HM . The role of the perichondrium in fetal bone development. Ann N Y Acad Sci. 2007;1116:59‐64. 18083921 10.1196/annals.1402.059

[11] Sharir A , Stern T , Rot C , Shahar R , Zelzer E . Muscle force regulates bone shaping for optimal load‐bearing capacity during embryogenesis. Development. 2011;138(15):3247‐3259. 21750035 10.1242/dev.063768

[12] Zimmermann EA , Riedel C , Schmidt FN , et al. Mechanical competence and bone quality develop during skeletal growth. J Bone Miner Res. 2019;34(8):1461‐1472. 30913317 10.1002/jbmr.3730

[13] Cooper KL , Oh S , Sung Y , Dasari RR , Kirschner MW , Tabin CJ . Multiple phases of chondrocyte enlargement underlie differences in skeletal proportions. Nature. 2013;495(7441):375‐378. 23485973 10.1038/nature11940 PMC3606657

[14] Maes C , Kobayashi T , Selig MK , et al. Osteoblast precursors, but not mature osteoblasts, move into developing and fractured bones along with invading blood vessels. Dev Cell. 2010;19(2):329‐344. 20708594 10.1016/j.devcel.2010.07.010 PMC3540406

[15] Isojima T , Sims NA . Cortical bone development, maintenance and porosity: genetic alterations in humans and mice influencing chondrocytes, osteoclasts, osteoblasts and osteocytes. Cell Mol Life Sci. 2021;78(15):5755‐5773. 34196732 10.1007/s00018-021-03884-w PMC11073036

[16] Piemontese M , Almeida M , Robling AG , et al. Old age causes de novo intracortical bone remodeling and porosity in mice. JCI Insight. 2017;2(17):e93771. 28878136 10.1172/jci.insight.93771 PMC5621920

[17] Kennedy OD , Herman BC , Laudier DM , Majeska RJ , Sun HB , Schaffler MB . Activation of resorption in fatigue‐loaded bone involves both apoptosis and active pro‐osteoclastogenic signaling by distinct osteocyte populations. Bone. 2012;50(5):1115‐1122. 22342796 10.1016/j.bone.2012.01.025 PMC3366436

[18] Jilka RL , Noble B , Weinstein RS . Osteocyte apoptosis. Bone. 2013;54(2):264‐271. 23238124 10.1016/j.bone.2012.11.038 PMC3624050

[19] Nakashima T , Hayashi M , Fukunaga T , et al. Evidence for osteocyte regulation of bone homeostasis through RANKL expression. Nat Med. 2011;17(10):1231‐1234. 21909105 10.1038/nm.2452

[20] Xiong J , Onal M , Jilka RL , Weinstein RS , Manolagas SC , O'Brien CA . Matrix‐embedded cells control osteoclast formation. Nat Med. 2011;17(10):1235‐1241. 21909103 10.1038/nm.2448 PMC3192296

[21] Kim HN , Xiong J , MacLeod RS , et al. Osteocyte RANKL is required for cortical bone loss with age and is induced by senescence. JCI Insight. 2020;5(19):e138815. 32870816 10.1172/jci.insight.138815 PMC7566701

[22] Hampel B , Malisan F , Niederegger H , Testi R , Jansen‐Dürr P . Differential regulation of apoptotic cell death in senescent human cells. Exp Gerontol. 2004;39(11–12):1713‐1721. 15582287 10.1016/j.exger.2004.05.010

[23] Jeon H , Boo YC . Senescent endothelial cells are prone to TNF‐α‐induced cell death due to expression of FAS receptor. Biochem Biophys Res Commun. 2013;438(2):277‐282. 23880344 10.1016/j.bbrc.2013.07.052

[24] Zheng H , Jiang L , Tsuduki T , Conrad M , Toyokuni S . Embryonal erythropoiesis and aging exploit ferroptosis. Redox Biol. 2021;48:102175. 34736120 10.1016/j.redox.2021.102175 PMC8577445

[25] Fali T , Fabre‐Mersseman V , Yamamoto T , et al. Elderly human hematopoietic progenitor cells express cellular senescence markers and are more susceptible to pyroptosis. JCI Insight. 2018;3(13):e95319. 29997288 10.1172/jci.insight.95319 PMC6124519

[26] Zhang L , Yang P , Chen J , et al. CD44 connects autophagy decline and ageing in the vascular endothelium. Nat Commun. 2023;14(1):5524. 37684253 10.1038/s41467-023-41346-y PMC10491636

[27] Arrázola MS , Lira M , Véliz‐Valverde F , et al. Necroptosis inhibition counteracts neurodegeneration, memory decline, and key hallmarks of aging, promoting brain rejuvenation. Aging Cell. 2023;22(5):e13814. 36973898 10.1111/acel.13814 PMC10186608

[28] Victorelli S , Salmonowicz H , Chapman J , et al. Apoptotic stress causes mtDNA release during senescence and drives the SASP. Nature. 2023;622(7983):627‐636. 37821702 10.1038/s41586-023-06621-4 PMC10584674

[29] Coradduzza D , Congiargiu A , Chen Z , Zinellu A , Carru C , Medici S . Ferroptosis and senescence: a systematic review. Int J Mol Sci. 2023;24(4):e95319. 10.3390/ijms24043658 PMC9963234 36835065

[30] Toyokuni S , Yanatori I , Kong Y , Zheng H , Motooka Y , Jiang L . Ferroptosis at the crossroads of infection, aging and cancer. Cancer Sci. 2020;111(8):2665‐2671. 32437084 10.1111/cas.14496 PMC7419040

[31] Zhou RP , Chen Y , Wei X , et al. Novel insights into ferroptosis: implications for age‐related diseases. Theranostics. 2020;10(26):11976‐11997. 33204324 10.7150/thno.50663 PMC7667696

[32] Wang L , Liu Y , Du T , et al. ATF3 promotes erastin‐induced ferroptosis by suppressing system Xc. Cell Death Differ. 2020;27(2):662‐675. 31273299 10.1038/s41418-019-0380-z PMC7206049

[33] Zhang C , Zhang X , Huang L , et al. ATF3 drives senescence by reconstructing accessible chromatin profiles. Aging Cell. 2021;20(3):e13315. 33539668 10.1111/acel.13315 PMC7963335

[34] McHugh J . Osteocyte death promotes bone loss. Nat Rev Rheumatol. 2020;16(10):539. 10.1038/s41584-020-0498-x 32843737

[35] Andreev D , Liu M , Weidner D , et al. Osteocyte necrosis triggers osteoclast‐mediated bone loss through macrophage‐inducible C‐type lectin. J Clin Invest. 2020;130(9):4811‐4830. 32773408 10.1172/JCI134214 PMC7456234

[36] Chang B , Liu X . Osteon: structure, turnover, and regeneration. Tissue Eng Part B Rev. 2022;28(2):261‐278. 33487116 10.1089/ten.teb.2020.0322 PMC9063188

[37] Bonewald LF . The role of the osteocyte in bone and nonbone disease. Endocrinol Metab Clin North Am. 2017;46(1):1‐18. 28131126 10.1016/j.ecl.2016.09.003 PMC5300041

[38] Masaldan S , Clatworthy SAS , Gamell C , et al. Iron accumulation in senescent cells is coupled with impaired ferritinophagy and inhibition of ferroptosis. Redox Biol. 2018;14:100‐115. 28888202 10.1016/j.redox.2017.08.015 PMC5596264

[39] Chen X , Li J , Kang R , Klionsky DJ , Tang D . Ferroptosis: machinery and regulation. Autophagy. 2021;17(9):2054‐2081. 32804006 10.1080/15548627.2020.1810918 PMC8496712

[40] Ingold I , Berndt C , Schmitt S , et al. Selenium utilization by GPX4 is required to prevent hydroperoxide‐induced ferroptosis. Cell. 2018;172(3):409.e421‐422.e421. 29290465 10.1016/j.cell.2017.11.048

[41] Ursini F , Maiorino M . Lipid peroxidation and ferroptosis: the role of GSH and GPx4. Free Radic Biol Med. 2020;152:175‐185. 32165281 10.1016/j.freeradbiomed.2020.02.027

[42] Zheng Y , Ritzenthaler JD , Burke TJ , Otero J , Roman J , Watson WH . Age‐dependent oxidation of extracellular cysteine/cystine redox state (E(h)(Cys/CySS)) in mouse lung fibroblasts is mediated by a decline in Slc7a11 expression. Free Radic Biol Med. 2018;118:13‐22. 29458149 10.1016/j.freeradbiomed.2018.02.026 PMC5884717

[43] Zhu B , Ni Y , Gong Y , et al. Formononetin ameliorates ferroptosis‐associated fibrosis in renal tubular epithelial cells and in mice with chronic kidney disease by suppressing the Smad3/ATF3/SLC7A11 signaling. Life Sci. 2023;315:121331. 36586573 10.1016/j.lfs.2022.121331

[44] Wu Q , Liang Z , Jiang J , et al. Macrophages originated IL‐33/ST2 inhibits ferroptosis in endometriosis via the ATF3/SLC7A11 axis. Cell Death Dis. 2023;14(10):668. 37816731 10.1038/s41419-023-06182-4 PMC10564909

[45] Fu D , Wang C , Yu L , Yu R . Induction of ferroptosis by ATF3 elevation alleviates cisplatin resistance in gastric cancer by restraining Nrf2/Keap1/xCT signaling. Cell Mol Biol Lett. 2021;26(1):26. 34098867 10.1186/s11658-021-00271-y PMC8186082

[46] Liu GH , Zhang DG , Lei XJ , et al. Effects of dietary selenium and oxidized fish oils on intestinal lipid metabolism and antioxidant responses of yellow catfish *Pelteobagrus fulvidraco* . Antioxidants (Basel). 2022;11(10):1904. 36290629 10.3390/antiox11101904 PMC9598306

[47] Magoro T , Dandekar A , Jennelle LT , et al. IL‐1β/TNF‐α/IL‐6 inflammatory cytokines promote STAT1‐dependent induction of CH25H in Zika virus‐infected human macrophages. J Biol Chem. 2019;294(40):14591‐14602. 31375561 10.1074/jbc.RA119.007555 PMC6779448

[48] Duan J , Li Z , Liu E , Long H , Chen L , Yang S . BSHXF‐medicated serum combined with ADSCs regulates the TGF‐β1/Smad pathway to repair oxidatively damaged NPCs and its component analysis. J Ethnopharmacol. 2023;316:116692. 37277086 10.1016/j.jep.2023.116692

[49] Taketani K , Kawauchi J , Tanaka‐Okamoto M , et al. Key role of ATF3 in p53‐dependent DR5 induction upon DNA damage of human colon cancer cells. Oncogene. 2012;31(17):2210‐2221. 21927023 10.1038/onc.2011.397

[50] Jiang Z , Qi G , He X , et al. Ferroptosis in osteocytes as a target for protection against postmenopausal osteoporosis. Adv Sci (Weinh). 2024;11(12):e2307388. 38233202 10.1002/advs.202307388 PMC10966575

[51] Xu P , Lin B , Deng X , Huang K , Zhang Y , Wang N . VDR activation attenuates osteoblastic ferroptosis and senescence by stimulating the Nrf2/GPX4 pathway in age‐related osteoporosis. Free Radic Biol Med. 2022;193(Pt 2):720‐735. 36402439 10.1016/j.freeradbiomed.2022.11.013

[52] Choi HE , Kim Y , Lee HJ , Cheon HG . Novel FoxO1 inhibitor, JY‐2, ameliorates palmitic acid‐induced lipotoxicity and gluconeogenesis in a murine model. Eur J Pharmacol. 2021;899:174011. 33705803 10.1016/j.ejphar.2021.174011

