# Supplementary material for: Osteocyte ferroptosis induced by ATF3/TFR1 contributes to cortical bone loss during ageing
Source: Cell Prolif. 2024 May 19;57(10):e13657. doi: 10.1111/cpr.13657 (PMC11471391; doi:10.1111/cpr.13657)
Supplement: Supplementary file 1 — Data S1. Supporting Information. [file CPR-57-e13657-s001.docx]

**Osteocyte ferroptosis induced by ATF3/TFR1 contributes to cortical bone loss during aging**

**Ying Yin^1, 2, 3, †^, Guang-Jin Chen^1, 2, 3, †^, Chen Yang^1, 2, 3^, Jia-Jia Wang^1, 2, 3^, Jin-Feng Peng^1, 2, 3^, Xiao-Fei Huang^1, 2, 3^, Qing-Ming Tang^1, 2, 3, *^, Li-Li Chen^1, 2, 3, *^**

^1^Department of Stomatology, Union Hospital, Tongji Medical College, Huazhong University of Science and Technology, Wuhan 430022, China

^2^School of Stomatology, Tongji Medical College, Huazhong University of Science and Technology, Wuhan 430030, China

^3^Hubei Province Key Laboratory of Oral and Maxillofacial Development and Regeneration, Wuhan 430022, China

**^†^**Ying Yin and Guangjin Chen contributed equally to this work.

**^*^Correspondence**:

Qingming Tang hust_tang@hust.edu.cn, Lili Chen [chenlili1030@hust.edu.cn](mailto:chenlili1030@hust.edu.cn)

**
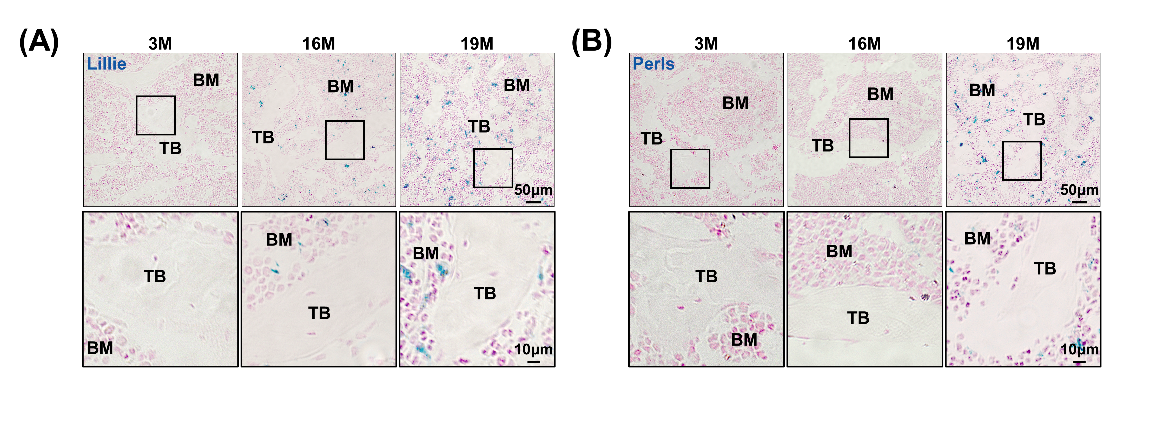
Supplementary Figures and Figure Legends**

**Figure S1**

No iron accumulation is observed in bone trabeculae. (**A**) Representative Lillie staining (blue) of the femur trabecular bone, showing ferrous iron content in the trabecular bone (n=3). (B) Representative Perl’s iron staining (blue) of the femur trabecular bone, showing the ferric iron content in the trabecular bone (n=3). TB, trabecular bone; BM, bone marrow.

**
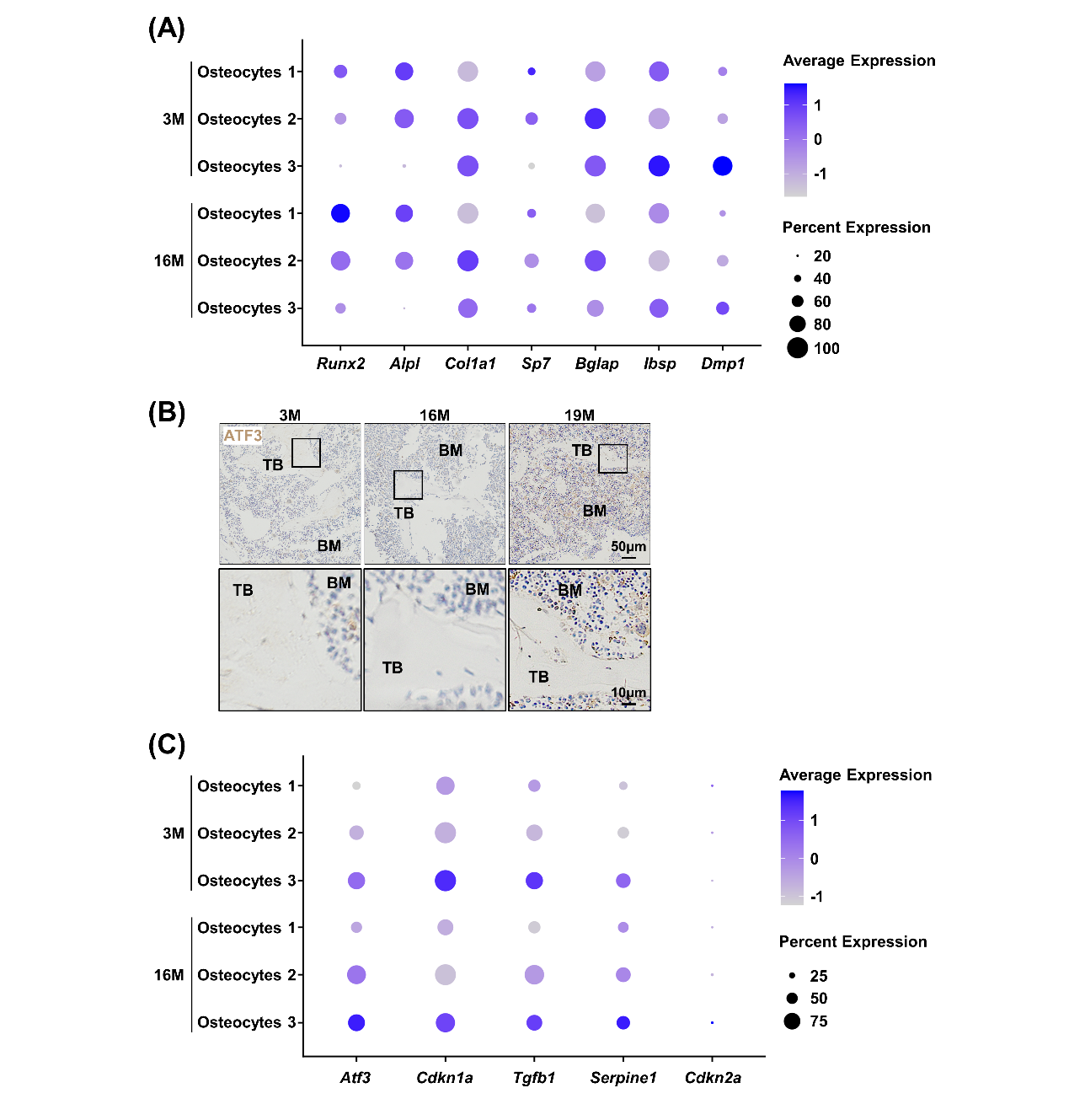
Figure S2**

Differences in gene expression among different osteocytes. (**A**) Dot plot showing osteogenesis-related genes of osteocytes 1, 2, and 3 in **Fig. 2A** from 3- and 16-month-old mice. (B) Immunohistochemical staining for ATF3 in femur trabecular bone of 3-, 16-, and 19-month-old mice (n=3). TB, trabecular bone; BM, bone marrow. (C) Dot plot showing Atf3 and aging-related genes of osteocytes 1, 2, and 3 in **Fig. 2A** from 3- and 16- month-old mice.

**
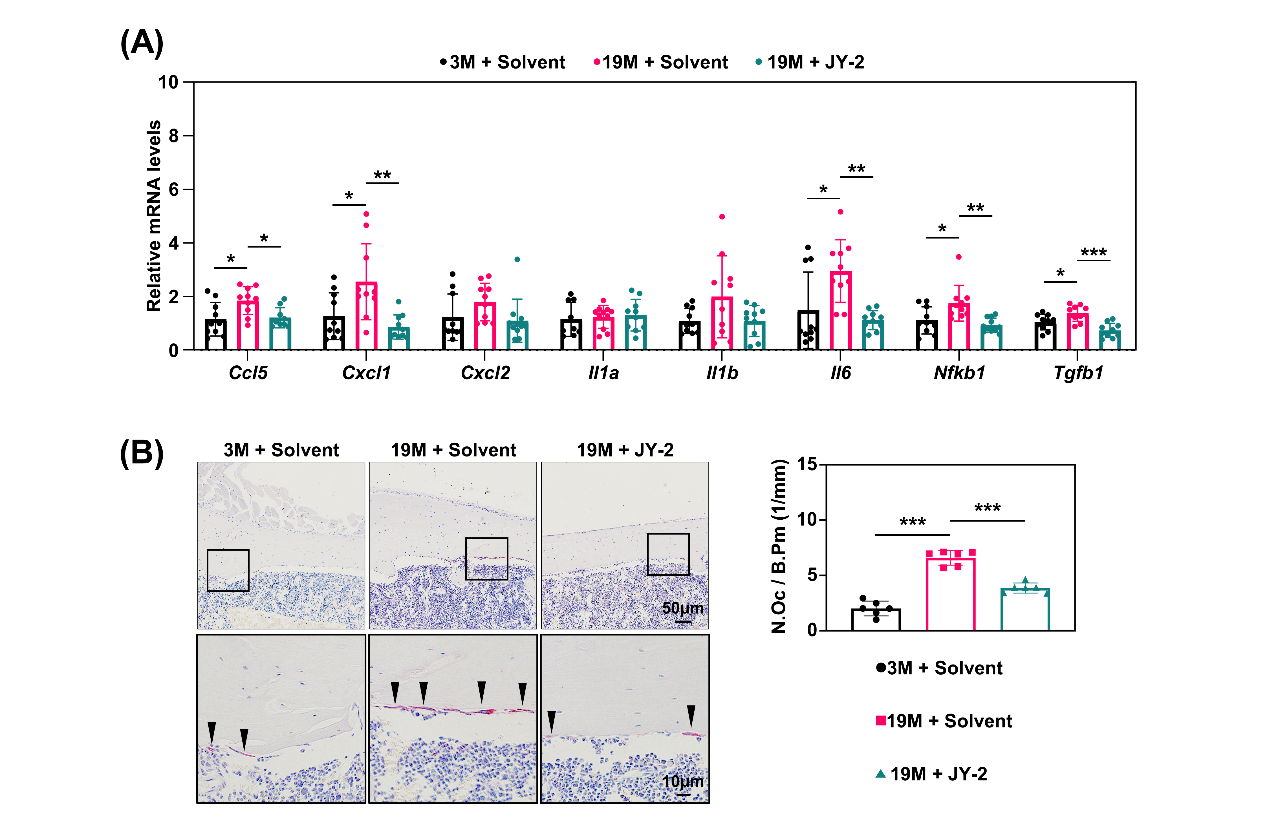
Figure S3**

Preventing ATF3 upregulation in aged mice alleviated SASP and bone resorption in cortical bone. (**A**) qRT-PCR analysis of the levels of *Ccl5*, *Cxcl1*, *Cxcl2*, *Il1a*, *Il1b*, *Il6*, *Nfkb1*, and *Tgfb1* in the femur cortical bone (n=3). (B) Representative images of TRAP staining of femur cortical bone with quantitative data at right. Black arrowheads point to osteoclasts. (n=3). *p < 0.05, **p < 0.01, ***p < 0.001.

**Supplementary Materials**

**Materials and methods**

**Animals**

Adult male C57BL/6J mice aged 3, 16, and 19 months used in this study were obtained from Beijing HFK Bioscience (Beijing, China) and fed antibiotic-free food and water ad libitum. The mice were randomly placed under a 12/12 h light-dark circle, with light from 8:00 a.m. In the in vivo experiments, JY-2 (MCE, HY-153347; 50 mg/kg, p.o., daily) was administered to the mice according to described experimental methods in the reported articles57. All animal experiments were approved by the Huazhong University of Science and Technology Laboratory Animal Centre and Use Committee (IACUC number: 3545).

**Micro-Computed Tomography (CT)**

After the femurs were harvested and fixed from the mice without soft tissues, the bone tissues were scanned at a resolution of 9 μm using Micro-CT (SkyScan 1176, Broker). The images were reconstructed and quantified using InstaRecon/NRecon Research Workplace software. The cortical width (Ct. Th) of the femurs was indexed to show differences among the cortical bones of the mice.

**Histology and Immunohistochemistry Staining**

After fixation with 4% paraformaldehyde and decalcification with 20% ethylenediaminetetraacetic acid solution, mice femora were made into paraffin sections with a thickness of 4 μm. Immunohistochemistry staining was performed on the paraffin sections using antibodies against GPX4 (Abclonal, A1933, 1:100), SLC7A11 (Proteintech, 26864-1-AP, 1:200), ATF3 (Bioss, bs-0519R, 1:100), EGR1 (Proteintech, 22008-1-AP, 1:200), and TFR1 (Proteintech, 10701-1-AP; dilution 1:200). Immunofluorescence was performed incubating antibody 488-conjugated P16 (Abcam, ab211542, 1:200) and ATF3 (Bioss, bs-0519R, 1:100) overnight at 4℃, and fluorescent secondary antibody (1:200) was then added for 1 h at room temperature. Osteocyte death in the cortical bone was assessed by TUNEL assays using One-step TUNEL Assay Kits (C1086, Beyotime, Shanghai, China), strictly according to the instructions. Images were acquired using a confocal microscope (Nikon-si-A1, Japan). Ferrous iron in the cortical bone was detected using Lillie’s Ferrous Iron Stain Kit (Solarbio, G3320), whereas ferric ions were detected using the Prussian Blue Iron Stain Kit (Solarbio, G1422), according to the manufacturer’s instructions. TRAP staining was performed on the standard protocols.

**Lipid Peroxide (LPO) Colorimetric Assay**

After the mice were sacrificed, the femora without soft tissue were harvested. The periosteum on the surface of the femur was scraped off, the bone marrow was removed, and the cortical bone tissue was put into phosphate-buffered solution for grinding at **−**10℃. The supernatant was then centrifuged, and LPO was detected according to the instructions of the Lipid Peroxide Colorimetric Assay Kit (Elabscience, E-BC-K176-M). Protein concentration was measured by the specifications of the Enhanced BCA Protein Assay Kit (Beyotime, P0010).

**Single-Cell Sequencing Analysis**

Single-cell sequencing data of the long bones of 3- and 16-month-old mice were obtained from the Gene Expression Omnibus (GSE145477) database. A subset function was then used to control the quality of each cell (nFeature_RNA > 200 & nFeature_RNA < 5000 & percentage mt <15). After processing the data using NormalizeData, the FindVariableFeature function was applied to screen feature genes for subsequent Principal Component Analysis (PCA). The first ten PCAs were selected to determine the cluster function. Dimensionality reduction analysis and cluster visualization were performed using the uniform manifold approximation and projection method. The FindAllMarkers function was used to identify the cell types in the clusters. Dentin matrix protein 1 has previously been reported as an osteocyte marker^1,2^. FindAllMarkers function was further used for identifying differentially expressed genes (DEGs) of 3- and 16-month-old mice osteocytes, displayed as a volcano plot (Log_2_[Flod change] ≥0.7, P ≤ 0.05). Twenty ferroptosis-related genes were selected from the DEGs using the FerrDb database (http://www. zhounan. org/ferrdb/current/). We then constructed and visualized the protein-protein-interacted network, which showed the hub differential ferroptosis-associated genes in osteocytes using Cytoscape software with the cytoHubba plugin (Maximal Clique Centrality method).

**MLOY4 Cell Culture, Viral Infection, and Treatments**

MLOY4 murine osteocytes (Sunncell, Wuhan, China) were cultured and passaged in DMEM supplemented with 10% (v/v) fetal bovine serum (Gibco, USA) and 1% penicillin-streptomycin (Biosharp, Beijing, China). For knockdown *Atf3* and *Tfr1*, siRNAs (Supplementary Table 1) were used according to the manufacturer’s protocols. For overexpression, MLOY4 cells were transfected with a lentiviral gene expression vector (3rd generation) containing *Slc7a11* (NM_ NM_011990.2; Supplementary Table 1). For inducing cell senescence, t-BHP (50 μM, Aladdin, Shanghai, China) was used to stimulate MLOY4 cells for 8 h followed by a series of cellular assays.

**Cell Staining**

To detect LPO in the MLOY4 cells, an LPO Fluorometric Assay Kit (Elabscience, E-BC-F003) was used in cell experiments according to the manufacturer’s instructions. Briefly, after the cells were transfected and stimulated with t-BHP for 8 h, the original medium was removed and a basal medium containing the probe with GFP was added. After 45 min of incubation at 37℃ in the dark, cells were nucleated using Hoechst (Beyotime, C1027) and incubated at 37℃ for 10 min in the dark to remove unbound dye and photographed using a confocal microscope. Additionally, MLOY4 cell death was assessed by TUNEL assays using One-step TUNEL Assay Kits (C1086, Beyotime, Shanghai, China) according to the manufacturer’s instructions, and images were obtained using a confocal microscope.

**Iron Measurement**

Intracellular ferrous and total iron were assessed separately using a Ferrous Ion Content (Solarbio, BC5415) and Total Iron Colorimetric (Elabscience, E-BC-K880-M) Assay Kits, respectively. After the indicated treatments, the MLOY4 cells were harvested and lysed in the indicated buffer. The whole-cell lysate was centrifuged at 12 000 × g for 10 min, and only the supernatant was used for the iron measurement assay. The experimental procedure strictly followed the manufacturer’s instructions.

**Immunoblotting**

Protein samples were obtained from MLOY4 cells or cortical bone tissues by ultrasonic oscillation or grinding with radioimmunoprecipitation assay lysate. As described previously, after denaturation in sodium dodecyl sulfate-polyacrylamide gel electrophoresis (SDS-PAGE) loading buffer, the protein extracts were fractionated on an SDS-PAGE gel and transferred onto polyvinylidene fluoride membranes^3^. The membrane was blocked and then incubated overnight with primary antibodies against ATF3 (Abcam, ab207434, 1:1000), SLC7A11 (Proteintech, 26864-1-AP, 1:1000), GPX4 (Abclonal, A21440, 1:500), TFR1 (Proteintech, 66180-1-Ig, 1:1000), FPN1 (Abclonal, A14885, 1:1000), STEAP3 (Abclonal, A0683, 1:500), and GAPDH (Proteintech, 10494-1-AP,1:10000). The following day, the membranes were incubated with secondary anti-rabbit antibodies (Santa Cruz Biotechnology Inc., 1:2000), followed by exposure to the Western Blotting Detection Kit (GE Healthcare, cat#: RPN2106).

**Quantitative Reverse Transcription Polymerase Chain Reaction (qRT-PCR) Analysis**

Total RNA was extracted from the cortical bone of the mice femurs after grinding in Trizol (Takara, Tokyo, Japan). Complementary DNA was synthesized using oligo-dT primers and reverse transcriptase (Takara Bio). Real-time RT-PCR was performed using the SYBR Green PCR protocol and BIO-RAD CFX Connect^TM^ Real-Time System (Bio-Rad Laboratories Inc., USA). Relative mRNA expression was normalized by GAPDH using the 2-△△Ct method. The primers used in these experiments are listed in Supplementary Table 1.

**Chromatin Immunoprecipitation (ChIP) assay**

Chromatin immunoprecipitation was performed according to the manufacturer’s instructions (Beyotime, ChIP Assay Kit, P2078). Antibodies against ATF3 (Abcam, ab207434) were used to pull down DNA from the MLOY4 cells. After purification, DNA was prepared for qRT-PCR amplification using these sequences (Supplementary Table 1).

**Luciferase Reporter Assay**

Luciferase assay experiments were performed on MLOY4 cells (1 × 105 cells/well in 24-well plates) as described previously, 375 ng firefly luciferase reporter vectors and 125 ng Renilla luciferase reporter vectors pGL-3.0 basic were co-transfected into cells using Lipo6000 (Beyotime) as instructed^4^. After 48 h, the cells were harvested. Firefly and Renilla luciferase activities were measured using a dual luciferase reporter assay system (Promega). Luciferase signals were standardized according to the firefly/Renilla ratio to confirm the transcriptional activity of *Tfr1* promoters.

**Statistical Analysis**

All data are displayed as means ± standard deviation. GraphPad Prism v. 9.0.2 was used for statistical analyses. Data were evaluated by unpaired two-tailed t-test or one-way analysis of variance and Tukey’s multiple comparison tests. All statistical analyses were performed using GraphPad Prism software version 9.0.2 (GraphPad Inc.). Statistical significance was considered significant at p < 0.05.

**Supplementary Tables and Associated Legends**

**Supplementary Table 1. Primer sequences of qRT-PCR, Chip assay, target genes knockdown, and overexpression.**

| Primers | Sequence (forward/reverse, 5'-3') | Usage |
| --- | --- | --- |
| *Gapdh* | TGTGTCCGTCGTGGATCTGA  TTGCTGTTGAAGTCGCAGGAG | qPCR |
| *Slc7a11* | GCTAACTGACTGCCCCTGGA  GCCACAACTGGCTTTCTGAC | qPCR |
| *Gpx4* | CCTGGACGCCAAAGTCCT  CCTTGGCTGAGAATTCGTGC | qPCR |
| *Casp3* | GAGCTTGGAACGGTACGCTA  CCGTACCAGAGCGAGATGAC | qPCR |
| *Casp6* | GAAGTGTTCGATCCAGCCGA  CAGGTTGTCTCTGTCTGCGT | qPCR |
| *Ripk3* | GCCTTCCTCTCAGTCCACAC  CTCACCAGAGGAACCGCATA | qPCR |
| *Tnf* | ACAGAAAGCATGATCCGCGA  GAGGCTGAGACATAGGCACC | qPCR |
| *Ar* | TATGGGGACATGCGTTTGGA  GGAGACGACAAGATGGGCAA | qPCR |
| *Atf3* | GTAGACCCTTCCCAGCCCTA  GTAGACCCTTCCCAGCCCTA | qPCR |
| *Egr1* | TGAGCATGACCAATCCTCCG  CAGGGATCATGGGAACCTGG | qPCR |
| *Epas1* | GTCTGGTAATGCCCCCATCT  ACTGCACAGGTCTACAGTGTC | qPCR |
| *Jun* | TGAGTGACCGCGACTTTTCA  GCATCGTCGTAGAAGGTCGT | qPCR |
| *Nedd4* | GCTTTTTCAACACCGCCGA  AGGGTTCCGACAGAGGGTAA | qPCR |
| *Nr4a1* | AGAGACGCGAGTGCAGC  CTTGAATACAGGGCATCTCCAC | qPCR |
| *Smad7* | ATTTTCTCAAACCAACTGCAGGC  AATTGAGCTGTCCGAGGCAA | qPCR |
| *Ubc* | GGTCAAACAGGAAGACAGACGTA  AGGGAAAACTAAGACACCTCCC | qPCR |
| *Zfp36* | GGACCTACTCAGAAAGCGGG  ACTTGTGGCAGAGTTCCGTT | qPCR |
| *Ccl5* | GCTGCTTTGCCTACCTCTCC  TCGAGTGACAAACACGACTGC | qPCR |
| *Cxcl1* | CTGGGATTCACCTCAAGAACATC  CAGGGTCAAGGCAAGCCTC | qPCR |
| *Cxcl2* | CCAACCACCAGGCTACAGG  GCGTCACACTCAAGCTCTG | qPCR |
| *Il1a* | CGCTTGAGTCGGCAAAGAAA  CTTCCCGTTGCTTGACGTTG | qPCR |
| *Il1b* | TGCCACCTTTTGACAGTGATG  AAGGTCCACGGGAAAGACAC | qPCR |
| *Il6* | GGAGCCCACCAAGAACGAT  TTGTGAAGTAGGGAAGGCCG | qPCR |
| *Nfkb1* | GCATCGTCCCAAAGGAGGAA  TCTGTGCGTGGCAACTACAT | qPCR |
| *Tgfb1* | GGAGAGCCCTGGATACCAACT  CCCGGGTTGTGTTGGTTGTA | qPCR |
| *Tfr1* #1 | AGATGGCCCAGTAAATACCTATGTT  ACAATGATGGCAGTTTGAAGTGAAG | Chip |
| *Tfr1* #2 | TTTGAGATGGAGGTTCTTGCTGTAC  CTGAGTCCCTGTAAGAGAAGCACAG | Chip |
| *Tfr1* #3 | AAGGCTGATACTTTCGAACTTGCAC  CCTCTGGATCTACGTCACTTCCTTC | Chip |
| si-*Atf3* #1 | CCUCCUGGGUCACUGGUAUTT  AUACCAGUGACCCAGGAGGTT | siRNA |
| si-*Atf3* #2 | GGAGGCGGCGAGAAAGAAATT  UUUCUUUCUCGCCGCCUCCTT | siRNA |
| si-*Tfr1* #1 | CCAGAUCAGCAUUCUCUAATT  UUAGAGAAUGCUGAUCUGGTT | siRNA |
| si-*Tfr1* #2 | GCUGGAACUUUCACAGAAUTT  AUUCUGUGAAAGUUCCAGCTT | siRNA |
| *Slc7a11* | GGAAGATCTGCCACCATGGTCAGAAAGCCAGTTGTGGCCACCATCT  CCGACGCGTTCATAATTCTTTAGAGTCTTCTGGTA | Overexpression |

**Reference:**

1. Lu Y, Xie Y, Zhang S, Dusevich V, Bonewald LF, Feng JQ. DMP1-targeted Cre expression in odontoblasts and osteocytes. *J Dent Res.* 2007;86(4):320-325.

2. Zhong L, Yao L, Tower RJ, et al. Single cell transcriptomics identifies a unique adipose lineage cell population that regulates bone marrow environment. *Elife.* 2020;9.

3. Chen G, Tang Q, Yu S, et al. Developmental growth plate cartilage formation suppressed by artificial light at night via inhibiting BMAL1-driven collagen hydroxylation. *Cell Death Differ.* 2023;30(6):1503-1516.

4. Tang Q, Xie M, Yu S, et al. Periodic Oxaliplatin Administration in Synergy with PER2-Mediated PCNA Transcription Repression Promotes Chronochemotherapeutic Efficacy of OSCC. *Adv Sci (Weinh).* 2019;6(21):1900667.
